# Associations Between Taq1A/C957T Polymorphic Variants and Autonomic Responsivity in a Slot Machine Task: Influence of Real-Life Gambling Exposure and Sex

**DOI:** 10.1007/s10899-025-10398-8

**Published:** 2025-05-30

**Authors:** Cathrine Hultman, Mattias Rehn, Guillaume Sescousse, Kent Nilsson, Sofia Vadlin, Cecilia Åslund

**Affiliations:** 1 Centre for Clinical Research, Västmanland Hospital Västerås, Region Västmanland, Uppsala University, Västerås, Sweden; 2 CHAP, Department of Public Health and Caring Sciences, Uppsala University, Uppsala, Sweden; 3 Lyon Neuroscience Research Center, INSERM U1028 - CNRS UMR5292, PSYR2 Team, University of Lyon, Lyon, France; 4 Department of Medical Sciences, Uppsala University, Uppsala, Sweden; 5 School of Health, Care and Social Welfare, Mälardalen University, Mälardalen, Sweden; 6 Department of Public Health and Caring Sciences, Uppsala University, Uppsala, Sweden

**Keywords:** Gambling, Skin conductance, Heart rate, C957T, Taq1A, cG×E

## Abstract

Monetary reward processing during gambling is associated with dopaminergic functioning. Emotional reactivity to different gambling stimuli can be indexed by autonomic nervous system (ANS) responses measured by skin conductance responses (SCR) and heart rate (HR). Genetic markers regulating neural dopaminergic activity, such as the D2 dopamine receptor, might confer differential sensitivity to gambling stimuli, which may also be modulated by previous exposure to gambling. To date, no previous studies have explored the relationship between genetic markers of the D2 dopamine receptor, real-life gambling exposure and ANS responses during gambling. Hence, this study explored associations and interactions between *DRD2* C957T (rs6277) and *ANKK1* Taq1A (rs1800497) genotypes, real-life gambling frequency and autonomic responses during reward anticipation and outcome delivery in a slot machine task producing wins, near-misses and full-misses. Participants (*n* = 270) performed a computerized slot machine task with recordings of SCRs and HR responses during gambling performance and provided saliva samples for DNA extraction. Taq1A A1 carriers showed increased SCRs and HR responses during reward anticipation and to wins. Greater responsivity during anticipation, as well as to wins and full-misses, was also observed in C957T heterozygotes. Regarding real-life gambling involvement, higher gambling frequency among Taq1A A1 carriers was associated with decreased HR responses during anticipation and to wins. Results suggest that polymorphic variants of the D2 dopamine receptor may confer differential sensitivity to different gambling stimuli which may further be modulated by real-life gambling exposures. However, further studies are needed in well powered samples of gamblers and control subjects.

## Introduction

For most consumers, gambling is enjoyable and harmless, but for others, it can become a compulsive and maladaptive activity comparable to drug addiction, and is thus classified as a behavioral addiction alongside “Substance-Related and Addictive Disorders” category in the DSM-5 (American Psychiatric Association, 2013). The addictive potential of gambling has been partly related to its ability to engage the neural reward system, especially the dopamine-rich region ventral striatum (Clark et al., 2019; de la Fuente-Fernández et al., 2002; Delgado et al., 2000; Joutsa et al., 2012; Linnet et al., 2010, 2011a, b, 2012; Reuter et al., 2005). The focus of the present study was to explore individual variability in emotional processing of different gambling cues based on dopaminergic genetic markers, and possible influence of previous exposure to gambling activities.

### The Role of Dopamine in Gambling

The role of dopamine and reward processing in problem gambling is an ongoing debate. Neuroimaging studies have reported mixed findings on whether reward processing among gamblers is expressed by *hyper-responsivity* or *hypo-responsivity*, i.e. heightened or decreased dopamine activity in the reward circuitry (Balodis et al., 2012; Luijten et al., 2017; van Holst et al., 2017; van Holst et al., 2012). Variability in dopaminergic genes modulates responsiveness of brain regions involved in reward processing through dopamine receptors (Foll et al., 2009), and studies have suggested a role of dopamine D2 receptors in problem gambling (Comings et al., 1996, 2001). However, association studies of pathological gambling and genotypes related to dopamine transmission have yielded mixed results (Comings et al., 2001; Gray & MacKillop, 2014; Lim et al., 2012; Lobo et al., 2010, 2015). In addition, several PET studies found no differences in baseline dopamine D2/D3 receptor binding between problem gamblers and controls but reported a relationship between reduced D2 receptor availability and mood-related impulsivity (Clark et al., 2012) and gambling severity (Boileau et al., 2013; Joutsa et al., 2012).

Hence, the role of dopamine in gambling appears more complex. Function of the dopaminergic system has been associated with reward prediction during anticipation, learning mechanisms and motivational aspects of gambling (Balodis & Potenza, 2020; Clark et al., 2019; Grant et al., 2016; Linnet, 2020; Zack et al., 2020). Dopamine neurons show differential phasic firing between predicted and unpredicted rewards and have been implicated in the anticipation of rewards (Fiorillo et al., 2003; Mikhael et al., 2022; Schultz, 1998, 2002, 2013). Influential gambling studies posit that the dopamine system in gamblers may be hyperreactive to gambling-specific cues rather than reward outcomes per se, leading to increased incentive value and motivation to gamble, through powerful expectancy effects (Clark et al., 2019; Linnet, 2014, 2020; Sescousse et al., 2013; Zack et al., 2020). Several studies report increased recruitment of the ventral striatum as well as cortical regions during anticipation of uncertain rewards and delivery of reward in gamblers (Knutson et al., 2001; Knutson et al., 2001; Knutson et al., 2003; Knutson & Greer, 2008; Linnet et al., 2012). However, contradicting results have also been reported (Luijten et al., 2017). These inconsistencies may partly stem from heterogeneity in terms of the tasks included (Clark, 2014; van Holst et al., 2017).

Although this may explain the process of increased attention and preoccupation in gambling (Clark et al., 2019), it does not fully explain why some individuals develop gambling problems, while others do not. Some theories suggest that biological factors may account for vulnerability toward engaging in addictive behaviors, which may be influenced by environmental factors, such as exposure to the addictive element (Leyton & Vezina, 2013, 2014). This may include experiences of emotional responses to gambling, i.e., certain genotypes might confer differential sensitivity to gambling stimuli.

#### ANKK1/DRD2

One of the most widely studied polymorphisms in addiction is the Taq1A (rs1800497) (Blum et al., 1995; Foll et al., 2009; Gorwood et al., 2012). This single nucleotide polymorphism (SNP) maps within the protein coding region in exon 8 of the ankyrin repeat and kinase domain containing 1 (*ANKK1*) gene located on chromosome 11 (Neville et al., 2004). The Taq1A causes an amino acid substitution resulting in either the minor A1 allele (thymine) or the A2 allele (cytosine) (rs1800497 - SNP - NCBI (nih.gov). The minor A1 allele has been associated with reduced dopamine D2 receptor availability in striatal regions (Gluskin & Mickey, 2016; Hirvonen et al., 2009; Jönsson et al., 1999; Noble et al., 1991; Pohjalainen et al., 1998; Ritchie & Noble, 2003; Thompson et al., 1997), as well as reduced reward responsivity (Cohen et al., 2005).

A linkage effect has been suggested between the Taq1A SNP and other functional polymorphisms which affect dopaminergic signaling, such as the synonymous polymorphism C957T (rs6277) located at exon 7 on the *DRD2* gene (Doehring et al., 2009; Hirvonen et al., 2009; Klaus et al., 2021; Ritchie & Noble, 2003). There is a discrepancy regarding the functionality of the allelic variants of this genotype. While Duan et al. (2003) found reduced mRNA stability and synthesis of the dopamine D2 receptor in T carriers, Hirvonen et al. (2004) and Hirvonen et al. (2005) conversely found reduced striatal D2 receptor availability in C carriers. Furthermore, Hirvonen et al. (2009) found that the C variant was associated with high extrastriatal DRD2 availability throughout the cortex and the thalamus, suggesting differential functional mechanisms of the C957T genotype in the cortex versus the striatum. Furthermore, some evidence suggests sex differences in D2 receptors, suggesting lower D2 dopamine-binding potentials among females. Pre-clinical studies have also proposed a role of estrogen in modulating dopamine release and increase in the expression of dopaminergic *DRD2* genes. However, sex-related modulations of human reward sensitivity are currently inconclusive [see Diekhof (2018) for a review].

Since both Taq1A and C957T appear to have effects on striatal dopamine D2 receptor availability, they both constitute functional markers for the D2 dopamine receptor. Few experimental studies have explored the relationship between these genotypes and gambling related behavioral measures. One study found increased reward related impulsiveness during acute stress among C957T C carriers (White et al., 2009). Another study found that both Taq1A A1 carriers and C957T C homozygotes showed better memory for rewarding stimuli during an incentive delay task followed by a delayed memory test (Richter et al., 2017). In addition, pathological gamblers carrying the Taq1A A1 allele displayed poorer performance in tasks measuring cognitive flexibility (Fagundo et al., 2014).

### Slot Machine Games in Experimental Research

The use of realistic and emotionally salient gambling games is central in experimental gambling research (Clark et al., 2019; Limbrick-Oldfield et al., 2017; Sescousse et al., 2013; van Holst et al., 2012). Slot machines are regarded as among the most addictive forms of gambling (Binde et al., 2017; Markham et al., 2015), mainly due to the distinguishing features associated with this type of gambling, such as high event frequency and duration, and intermittent random reward delivery producing maximum outcome uncertainty. Slot machine gambling comprise distinct cognitive elements including an anticipation phase where the reels spin, and the outcome phase where the reels stop to reveal the outcome (Griffiths & Auer, 2013; Murch & Clark, 2016; Zack et al., 2020). In addition to anticipation and reward processing, slot machines also comprise other features such as near-misses, which constitute one of the most studied structural characteristics in games of chance. These are non-win outcomes that fall just short of a winning outcome. Evidence suggests that near-misses facilitate persistent play by manipulating players’ perceptions of winning and sense of control (Billieux et al., 2012; Clark et al., 2009, 2012a; Qi et al., 2011). This is supported by studies reporting that near-misses have been associated with increased psychophysiological arousal (Clark et al., 2012a; Dixon et al., 2011; Hultman et al., 2022), and prolonged gambling sessions (Cote et al., 2003; Kassinove & Schare, 2001; MacLin et al., 2007). Moreover, brain imaging studies show heightened striatal and insular recruitment to near-misses in both gamblers and non-gamblers (suggesting increased activity in dopamine-rich areas) and greater reward expectancy relative to full-misses (Chase & Clark, 2010; Clark et al., 2009; Dymond et al., 2014; Sescousse et al., 2016; Worhunsky et al., 2014). Dopaminergic function associated with a variety of perceptual constructs in gambling, including near-misses, are less studied in humans. Therefore, inherent genetic dopaminergic functions in relation to emotional responsivity to anticipation, wins, near-misses and losses in the context of slot machine gambling may provide further insights into interindividual differences in affective processing during gambling.

### Autonomic Nervous System Responses During Gambling

Brain imaging techniques have been widely used to study reward responses in relation to neural functioning. Another, less invasive approach, to capture such response during gambling is through psychophysiological measures of the autonomic nervous system (ANS). Phasic measures of heart-rate (HR) and skin conductance responses (SCRs) are considered reliable and complementary measures of event-related autonomic responses to reward stimuli in experimental gambling settings (Lole et al., 2012, 2014). Several studies report increases in autonomic arousal to rewards (Coventry & Constable, 1999; Coventry & Hudson, 2001; Diskin & Hodgins, 2003; Moodie & Finnigan, 2005; Sharpe, 2004; Wilkes et al., 2010; Wulfert et al., 2005, 2008), as well as elevated SCRs and HR responses to near-misses relative to full-misses (Clark et al., 2012a; Dixon et al., 2011; Hultman et al., 2022). Thus, autonomic activity, measured by SCRs and HR provides objective indicators of emotional reactivity to different gambling stimuli. Furthermore, while sex differences in neural reward processing have been widely explored, studies investigating sex differences in terms of ANS responses to gambling related cues are scarce. Some early studies reported no differences in males and females during gambling (Coventry & Hudson, 2001; Wulfert et al., 2008), while one recent study from our group reported larger SCRs to wins in females during slot machine play (Hultman et al., 2022).

To date, no studies have investigated the potential relationship between D2 dopamine receptor polymorphisms and ANS responses during gambling. Given the implications of dopaminergic activity during gambling and individual variability in reward processing, interactions of inherent genetic markers and more peripheral physiological ANS measures of cue-reactivity (considered indexes of emotional processing) may provide further insight into attentional and motivational processing during gambling, potentially underlying individual risks of developing problem gambling. Specifically, D2 dopamine receptor genotypes might confer differential ANS response sensitivity to gambling stimuli and may also be modulated by previous exposure to gambling activities.

Given the potential sex-related effects on dopaminergic and ANS activity, differential sensitivity to gambling stimuli as a function of genotype may also be modulated by sex.

### Aim

The aim of this study was to explore the possible influence of single nucleotide polymorphic variants of the dopaminergic *DRD2* and *ANKK1* genotypes in relation to autonomic responses (SCR and HR) during anticipation and outcomes on a simulated slot machine game delivering unpredictable wins, near-misses and full-misses, in a community sample of young adults. A second aim was to investigate candidate gene-environment interaction (cG×E) effects of genotypes and real-life gambling frequency on autonomic responses during slot machine gambling, further testing for possible sex differences.

## Method

### Participants

Participants were recruited from a large longitudinal cohort study of young adults born in 1997 and 1999 (Survey of Adolescent Life in Västmanland, SALVe Cohort; the 2015 wave 2 [*n* = 1644]). Vadlin et al. (2018), for a full report on the cohort wave 2 inclusion. Data were collected during an experimental session at Västmanland County Hospital, Västerås, Sweden. Initially, a subset of participants of the cohort were included based on previous gambling experience, to maximize the number of gamblers in the study. Cohort participants who had previously provided a saliva sample for DNA extraction and participated in 2015 wave 2 were eligible for inclusion. They were then randomized, stratified based on age and sex and consecutively included to the experimental study. Volunteer participants were recruited between 2017 and 2019. A final sample of 270 volunteers (140 females, 130 males) were included in the study (age 18–22 years) [see Hultman et al. (2022), for a full report on the inclusion]. None of the participants reported any current or previous history of gambling disorder diagnosis. The level of problem gambling was assessed with the Problem Gambling Severity Index (PGSI) (Ferris & Wynne, 2001), a 9-question screening tool with possible scores between 0 and 27. Based on self-reports on the PGSI, 35 participants fell in the categories from low-risk to problem gambling (Table 1) (Public Health Agency of Sweden, 2010). Participants also reported gambling frequency with different forms of gambling during the past 12 months via 4 items (During the past 12 months, how often have you gambled with money on…? 1) online poker, casino or similar?; 2) poker, casino or similar, but not online?; 3) electronic gambling machines/slot machines, but not online?; 4) sports betting?), with choice categories for each question, ranging between 0 and 8. Scores were then summarized from each question to index the total level of gambling frequency (range: 0–32). Level of problem gambling based on self-reported PGSI scores and gambling frequency summary scores are presented in Table 1. The sub-sample of the experimental study was compared to the larger cohort in terms of socioeconomic status (parents’ monthly income) and origin (parents born in/outside Scandinavia) using independent samples t-tests, revealing no significant differences between the samples (*p* = 0.690, *p* = 0.893 respectively). This study was approved by the Ethical Review Board of Uppsala (dnr 2016/569), with an extended approval (dnr: 2019 − 01368). Detailed information on the procedure was given by the examiner and all participants provided informed consent upon arrival to the experimental session.

**Table 1:** Level of problem gambling (PGSI) and previous gambling experience including forms of gambling, distributed among males and females

	Range	Mean (SD)	*N* (%)	Males (%)	Females (%)
PGSI Total score	0–27	0.13 (0.34)			
Score ≥ 1			35 (13.0%)	30 (23.1%)	5 (3.6%)
Levels*					
*Non-problem gambler* *Low-risk gambler* *Moderate-risk gambler* *Problem gambler*			235 (87.0%) 20 (7.4%) 14 (5.2%) 1 (0.4%)	100 (76.9%) 18 (13.8%) 11 (8.5%) 1 (0.8%)	135 (96.4%) 2 (1.4%) 3 (2.1%)-
**Previous gambling experience** ***Total score***	0–32	1.0 (2.1)			
Score (≥ 1)			84 (31.1%)	65 (50.0%)	19 (13.6%)
***Online casino*** Score (≥ 1)	0–8	0.34 (0.99)	41 (15.2%)	35 (26.9%)	6 (4.3%)
***Land-based casino*** Score (≥ 1)	0–8	0.21 (0.53)	43 (15.9%)	40 (30.8%)	3 (2.1%)
***Land-based EGM*** Score (≥ 1)	0–8	0.06 (0.26)	16 (5.9%)	10 (7.7%)	6 (4.3%)
***Sports betting*** Score (≥ 1)	0–8	0.38 (1.02)	47 (17.4%)	38 (29.2%)	9 (6.4%)
**Total number of participants**			270 (100%)	130 (100%)	140 (100%)

*PGSI cut-off scores: 0 = non-problem gambler, 1–2 = low-risk gambler, 3–7 = moderate-risk gambler, ≥ 8 = problem gambler (Public Health Agency of Sweden, 2010)

### Procedure

Participants performed several computerized cognitive and gambling tasks during individual experimental sessions, along with a web-based questionnaire including questions on problem gambling (PGSI) and gambling frequency. The present study focuses on slot machine gambling and uses data originally reported in a previous study comparing levels of ANS responses to different outcomes during a Slot Machine Task with simultaneous psychophysiological recordings of electrodermal activity (EDA) and electrocardiograms (ECG) (Hultman et al., 2022). The current study extends the previous one by investigating whether ANS responses differ depending on genotypes. The sensory data was recorded with Biopac System MP150 (Biopac Systems, Goleta, CA, USA). The computer from which the tasks were performed was connected to the Biopac system and to a second computer running *Acqknowledge* v5.0.1 to event-mark the psychophysiological data through digital channels. The sensory equipment for the skin conductance recordings were two grounded Ag–AgCl electrodes (Biopac EL507 with a BN-PPGED amplifier module, sample rate 62.5, constant voltage 0.5 V, low-pass filter 3.0 Hz, high-pass filter DC). Electrodes were attached to the thenar and hypothenar eminences of the non-dominant hand, and a 0.05 M NaCl electrolyte paste GEL101 was used. The ECG sensory equipment were three disposable grounded Ag–AgCl electrodes (Biopac EL504, with a BN-RSPEC module, sample rate 2000, low-pass filter 35 Hz, high-pass filter 1 Hz). Electrodes were attached to the right shoulder and grounded to the eighth rib on the left and right side and contained liquid hydrogel (4% NaCl). In *Acqknowledge*, the EDA signal was transformed into micro siemens (µS), and the ECG signal was converted into heart rate (HR) in beats per minute (BPM). Despite efforts to regulate room temperature and humidity the room temperature ranged from 20 °C to 32 °C (M = 25.5 °C) and the humidity from 20 to 58% (M = 41%) across the sessions, due to seasonal changes (Detailed description of the procedure is provided by Hultman et al. (2022). Participants received a gift card of 1000 SEK (≈ 100 €) for participation in the entire session, with the possibility of additional compensation depending on their performance on the gambling task.

### Slot Machine Gambling Task

**Fig. 1:**
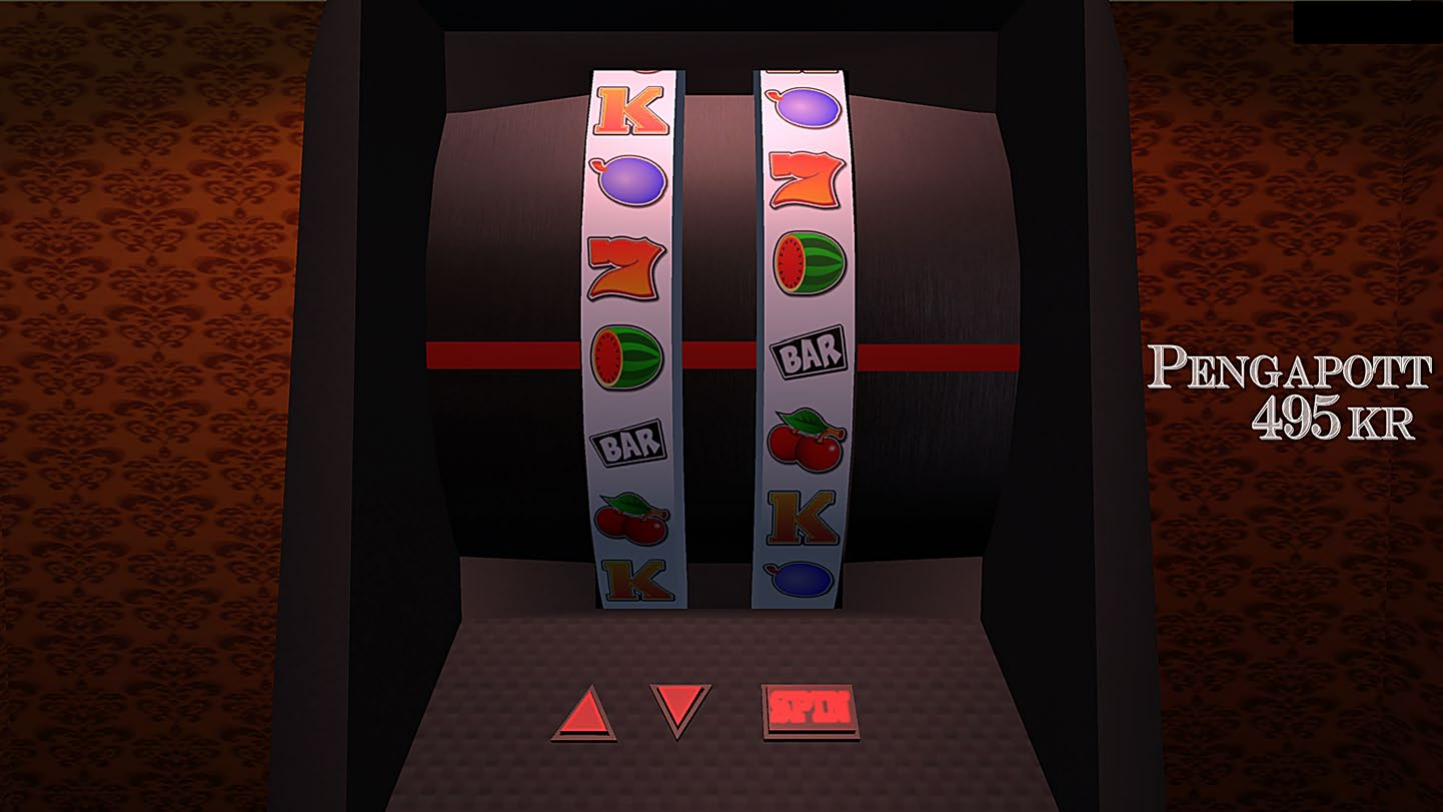
Screen display of the computerized slot machine gambling task

Participants performed a modified version of a computerized Slot Machine Gambling Task (Sescousse et al., 2016) previously described by Hultman et al. (2022). Graphics and sounds were modified to resemble an internet casino environment (Fig. 1). The task displays two reels showing six symbols and a red payline placed horizontally across the center. Starting each trial participants scrolled the left reel to choose one of the six symbols and then spun the right reel. The reel spun for 3 s and then slowly decelerated to a standstill during an interval of 2.8–6.0 s, resulting in a total anticipation interval of 5.8–9.0 s. If two matching symbols aligned on the payline, a ‘win’ was delivered and followed by a short melody, applause, and the messages “Jackpot!” (5 s) and “You won 100 SEK” (4 s) displayed on the screen. The task also generated ‘near-miss’ outcomes where the matching symbol stopped one position before or after the payline. All other outcomes were termed ‘full-misses’. All non-win outcomes were followed by a sound signifying a loss and the message “No win” (4 s). Unknown to the participants, the task was standardized with a fixed distribution and order of different gambling outcomes, giving 10 ‘wins’, 20 ‘near-misses’ and 30 ‘full-misses’, across the entire task. Ratings of satisfaction, motivation, and perceived chance of winning were also obtained with three questions following each trial, but these are not analyzed in the current study. Participants started the task with a loan of 500 SEK (≈ 50 €) to gamble with. Each trial involved a bet of 15 SEK (≈ 1.5 €), and a winning outcome resulted in an additional 100 SEK (≈ 10 €). The task started with three practice trials followed by the main session with 60 trials. The standardized distribution of outcomes resulted in an end balance of 750 SEK (≈ 75 €). Thus, each participant received the maximum gratification; a gift card of 200 SEK (≈ 20 €).

### Genotyping

Saliva samples (200 µL) for the genotyping procedure were collected using the Oragene^®^ DNA self-collection kit (Ottawa, Ontario, Canada) using a silica-based extraction method (Kleargene™, LGC, Biosearch Technologies). Genotyping analyses of all the SNPs (*rs1800497* A2 > A1 and *rs6277*C > T) were performed using the Kbioscience Allele-Specific Polymorphism assay based on competitive allele-specific PCR and bi-allelic scoring (LGC^®^, England). Allele discrimination was completed using SNPviewer^®^. Genotype frequency and distribution are presented in Table 2.

**Table 2:** Descriptives of genotype distributions

Gene	Polymorphism	SNP	Minor allele	Genotypes	*N*	Hardy-Weinberg equilibrium
*ANKK1*	Taq1A	rs1800497	A1	A1/A1 A1/A2 A2/A2	13 94 160	0.865
*DRD2*	C957T	rs6277	T	T/T T/C C/C	66 132 70	0.810

### Data Processing and Analysis

Data inspection and management of the psychophysiological data was conducted prior to statistical analysis as described by Hultman et al. (2022). Initially, the recoded data was inspected to identify and remove recoding artifacts in the EDA and ECG signals attributable to recording noise, excessive movement, or electrode detachment. Subsequently, there was a quality assessment of the psychophysiological data leading to the exclusion of 69 participants from the SCR analysis, who had missing data due to technical failure in the recording equipment. Two participants with heart arrythmia, and four outliers with high BPM compared to the group mean (> 2.5 SD) were excluded. According to self-reports 14 participants used antidepressants (such as selective serotonin reuptake inhibitors; serotonin, and norepinephrine reuptake inhibitors; tetracyclic piperazine-azepine; and/or bupropion). These were excluded due to possible confounding effects on dopaminergic signaling and potentially automatic processing through SCRs and HR. Thus, a total of 190 participants were included in the SCR analysis and 251 participants in the HR analysis. The data was exported to MATLAB for analysis, using the *Ledalab* software package (www.ledalab.de). Event-related sympathetic activity was extracted using a continuous decomposition analysis of the EDA signal.

SCRs were defined as the maximum SCR amplitude within 1–4 s post gambling stimulus onset, minus the 1 s pre gambling stimulus onset baseline value. The minimum amplitude criterion for SCRs was set at 0.05 µS (Boucsein, 2012). SCR amplitudes were calculated for: stimuli onset of the anticipation response (pressing the SPIN-button), and stimuli onset for wins, near-misses and losses (the reel stops). The HR response was calculated in beats per minute (BPM) for every half second during 0–8 s post gambling stimulus onset. A biphasic cardio-vascular response, with an initial deceleration and subsequent acceleration responses was calculated based on (Hodes et al., 1985): baseline value (average HR 1 s pre-stimulus), deceleration component (the minimum 0–3 s post-stimulus value, minus the baseline value), and acceleration component (the maximum 2–6 s post-stimulus value, minus the deceleration component).

Statistical analyses were performed using IBM SPSS Statistics version 28. Linear regression analyses using Generalized Linear Models (GLM) were conducted. In all post-hoc comparisons the minor and heterozygote alleles were compared against the major allele of each gene. Initially, separate analyses of main effects of genotypes on ANS responses were conducted, using 3 ANS measures (SCRs, HR acceleration, HR deceleration) and 4 stimuli conditions (anticipation, wins, near-misses and full-misses) as dependent outcome variables resulting in 12 separate analyses for each gene. Analyses of main effects were also adjusted for “sex” and “gambling frequency”. Next, two-way interaction models were conducted to analyze interaction between genotype and (1) “sex” (genotype*sex), and (2) gambling frequency (genotype*gambling frequency). To explore the effects of genotypes, all analyses were first run with three groups of homozygous major, heterozygous, and homozygous minor alleles. To improve power, particularly in the interaction analyses, the effect of homozygotes for the major allele versus *presence of the minor allele* was also tested by combining homozygotes for the minor allele and heterozygotes (Taq1A = A2:A2 vs. A1:A1/A1:A2, C957T = C: C vs. T: T/T: C). A significance threshold of 0.05 was used.

## Results

Allele frequencies did not deviate from Hardy-Weinberg equilibrium on Taq1A or C957T in our sample (Table 2). The allele frequencies of each genotype were also compared in terms of sex, with no statistical differences between males and females (*p* = 0.080, *p* = 0.565 respectively). Independent samples t-test revealed significant sex differences in level of gambling frequency (t(150.68) = -6.403, *p* < 0.001), with higher scores among males (*M =* 1.81, *SD* = 2.69) than females (*M* = 0.24, *SD* = 0.81).

### Association Between Gambling Frequency and ANS Responses to Slot Machine Stimuli

Linear regression analyses using Generalized Linear Models were conducted to investigate associations between gambling frequency and ANS responses (SCRs, HR deceleration and acceleration) to different slot machine stimuli. The model revealed a significant effect of gambling frequency on SCR responses to wins, specifically a higher level of gambling exposure was associated with smaller SCR responses to winning outcomes (*B* = -0.075, *CI* = -0.138– -0.011, *p* = 0.021). There were no associations between gambling frequency and SCR responses during anticipation or to near-misses or full-misses.

There were no significant associations between gambling frequency and HR acceleration to any gambling stimuli. However, the model showed an effect of gambling frequency on HR deceleration to full-misses, where a higher level of gambling frequency was associated with smaller HR deceleration to full-misses (*B*= -0.124, *CI* = -0.226– -0.022, *p* = 0.017).

### Association Between Genotypes and Autonomic Responses to Slot Machine Stimuli

A linear regression analysis using Generalized Linear Models was conducted to investigate the association between dopaminergic genotypes (Taq1A/rs1800497 and C957T/rs6277) and ANS responses (SCRs, HR deceleration and acceleration) to different slot machine stimuli.

#### *ANKK1*, Taq1A (rs1800497)

Results did not show a significant association between Taq1A (rs1800497) and anticipatory SCRs while adjusting for “sex” and “gambling frequency”. However, an association was revealed when testing the effect of homozygotes for the major allele (A2:A2) versus presence of the minor allele (A1:A1/A1:A2), where A1:A1/A1:A2 carriers had larger anticipatory SCRs than A2:A2 carriers (*B* = 0.165, *CI* = 0.028–0.302, *p* = 0.018) (Table 3).

**Table 3:** Linear regression (GLM) analyzing main effects of genotypes on SCR responses to different gambling stimuli (anticipation, wins, near-misses and full-misses)

Skin conductance responses (SCRs)			
	Model effect (*p*)	B (CI)	*p*
**Taq1A**			
Anticipation	0.059		
*A1/A1*		0.20 (-0.13–0.52)	0.239
*A1/A2*		0.16 (0.02–0.30)	**0.026**
*A2/A2*		0^a^	
Wins	**0.004**		
*A1/A1*		0.68 (0.18–1.18)	**0.008**
*A1/A2*		0.27 (0.05–0.49)	**0.015**
*A2/A2*		0^a^	
Near-misses	0.493		
*A1/A1*		0.12 (-0.14–0.38)	0.359
*A1/A2*		0.05 (-0.06–0.17)	0.370
*A2/A2*		0^a^	
Full-misses	0.256		
*A1/A1*		0.16 (-0.06–0.38)	0.146
*A1/A2*		0.05 (-0.05–0.15)	0.311
*A2/A2*		0^a^	
**C957T**			
Anticipation	**0.007**		
*T/T*		0.00 (-0.18–0.19)	0.972
*C/T*		0.22 (0.05–0.38)	**0.009**
*C/C*		0^a^	
Wins	0.133		
*T/T*		-0.04 (-0.33–0.26)	0.808
*C/T*		0.20 (-0.06–0.45)	0.136
*C/C*		0^a^	
Near-misses	0.095		
*T/T*		0.02 (-0.13–0.17)	0.809
*C/T*		0.13 (-0.004–0.26)	0.057
*C/C*		0^a^	
Full-misses	**0.001**		
*T/T*		0.04 (-0.09–0.16)	0.578
*C/T*		0.18 (0.07–0.29)	**< 0.001**
*C/C*		0^a^	

*p* < 0.005. *B =* regression coefficient. *CI =* 95% Wald confidence interval. 0^a^ = reference allele

Analyses also showed a significant relationship between Taq1A variants and SCRs to wins (*p* = 0.004), specifically revealing larger responses for A1:A1 carriers than A2:A2 carriers (*p* = 0.008) (Table 3). Analyzing the effect of homozygotes for the major allele versus presence of the minor allele also revealed larger SCRs for A1:A1/A1:A2 carriers than A2:A2 carriers (*p* = 0.003) (Table 3).

There were no significant associations between Taq1A variants and changes in SCRs to near-misses or full-misses. Also, there were no significant associations between Taq1A variants and HR deceleration or acceleration to any gambling stimuli.

#### *DRD2*, C957T (rs6277)

Analysis showed an association between C957T (rs6277) and anticipatory SCRs (*p* = 0.007). Specifically, T:C allele carriers were associated with larger anticipatory SCRs than C: C carriers (*p* = 0.009) (Table 3). Furthermore, after analyzing the effect of homozygotes for the major allele (C: C) versus presence of the minor allele (T: T/C: T), no significant associations were seen. No significant associations were shown between C957T variants and changes in SCRs to wins or near-misses.

Analyses further revealed a significant relationship between the C957T variants and SCR responses to full-misses (*p* = 0.001). Specifically, T:C allele carriers were associated with larger responses than C: C carriers (*p* < 0.001). Furthermore, analyzing the effect of the major allele versus presence of the minor allele showed larger SCRs to full-misses among T: T/C: T carriers than C: C carriers (*B* = 0.131, *CI* = 0.028–0.233, *p* = 0.013) (Table 3).

There were no significant associations between C957T and HR deceleration or acceleration to any gambling stimuli.

### Interactions Between Genotypes and Sex on ANS Responses To Slot Machine Stimuli

Two-way interactions using Generalized Linear Models was conducted to investigate interactions between dopaminergic genotypes (Taq1A/rs1800497 and C957T/rs6277) and sex on ANS responses (SCRs, HR deceleration and acceleration) to different slot machine stimuli.

#### *ANNK1*, Taq1A (rs1800497)

**Fig. 2:**
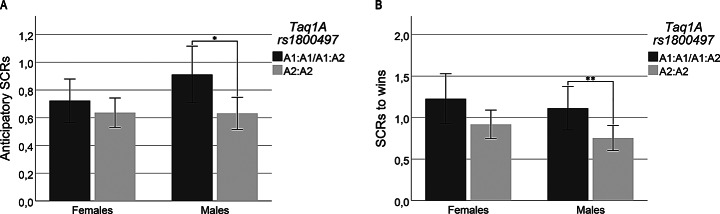
Illustration of sex differences in SCRs by genotype variants. **A**) SCRs during anticipation among Taq1A variants clustered by sex. **B**) SCRs to wins among Taq1A variants clustered by sex. *Error bars*: 95% CI

Two-way interaction analyses showed no significant interactions between Taq1A variants and sex on anticipatory SCRs. However, an interaction was shown when testing effects of homozygotes for the major allele versus presence of the minor allele (*p* = 0.027). Subgroup analyses divided by sex revealed larger anticipatory SCRs among male A1:A1/A1:A2 carriers compared to male A2:A2 carriers (*B* = 0.288, *CI* = 0.061–0.515, *p* = 0.013), but no significant differences among females were detected (Fig. 2A).

There was also an interaction between Taq1A variants and sex on SCRs to wins (*p* = 0.004). Subgroup analyses divided by sex further showed significantly larger SCRs to wins in A1:A1 carriers than A2:A2 carriers in both females and males (*B* = 1.390, *CI* = 0.271–2.508, *p* = 0.015, *B =* 0.539, *CI* = 0.045–1.034, *p* = 0.033, respectively). However, male heterozygotes also showed larger SCRs to wins compared to A2:A2 carriers (*B* = 0.313, *CI* = 0.023–0.603, *p* = 0.035), but such differences were not significant among females. An interaction was also revealed when analyzing the effect of homozygotes for the major allele versus presence of the minor allele (*p* = 0.008). Subgroup analysis showed significantly larger SCRs to wins in A1:A1/A1:A2s compared to A2:A2 among males (*B* = 0.360, *CI* = 0.089–0.630, *p* = 0.009), but such differences were not significant among females (Fig. 2B). Figure 2A and B illustrates the level of SCRs for *combined groups* of Taq1A alleles, clustered by sex.

There were no significant interactions between Taq1A variants and sex regarding changes in SCRs to near-misses or full-misses. There were no significant interactions between Taq1A variants and sex regarding HR deceleration or acceleration to any gambling stimuli.

#### *DRD2*, C957T (rs6277)

**Fig. 3:**
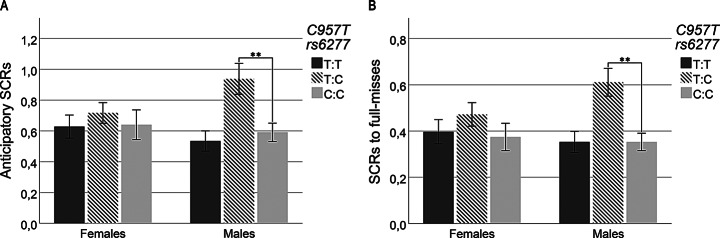
Illustration of sex differences in SCRs by genotype variants. **A**) SCRs during anticipation among C957T variants clustered by sex. **B**) SCRs to wins among C957T variants clustered by sex. *Error bars*: 95% CI

Two-way interactions were revealed between C957T variants and sex on anticipatory SCRs (*p* = 0.007). Subgroup analyses showed significantly larger responses in male heterozygotes compared to male C: Cs (*B* = 0.347, *CI* = 0.114–0.581, *p* = 0.004), while such differences were not significant in females (Fig. 3A). However, no interactions were seen when comparing the major allele versus the presence of the minor allele. There were no significant interactions between C957T variants and sex regarding changes in SCRs to wins or near-misses.

There was also an interaction between C957T variants and sex on SCRs to full-misses (*p =* 0.003). Subgroup analysis showed significant differences among males in the subgroup analysis, with larger SCRs to full-misses in male heterozygotes compared to male C: C carriers (*B* = 0.347, *CI* = 0.114–0.581, *p* = 0.004), while such differences were not significant in females (Fig. 3B). An interaction was also shown when comparing the major allele versus the presence of the minor allele (*p* = 0.003). However, subgroup analysis failed to detect any differences in males and females separately. Figure 3A and B illustrates the effect of C957T heterozygotes by displaying SCRs for *three alleles*, clustered by sex.

There were no significant interactions between C957T variants and sex regarding HR deceleration or acceleration to any gambling stimuli.

### cG×E of Genotypes and Gambling Frequency on Autonomic Responses to Slot Machine Stimuli

A linear regression analysis using Generalized Linear Models was used to investigate cG×E effects of genotypes (Taq1A/rs1800497 and C957T/rs6277) and gambling frequency on ANS responses (SCRs, HR deceleration and acceleration) to different slot machine stimuli.

#### *ANKK1*, Taq1A (rs1800497)

There were no significant interactions between Taq1A (rs1800497) variants and gambling frequency on SCRs or HR acceleration responses during anticipation. Also, there were no significant interactions between Taq1A variants and gambling frequency on SCRs responses to slot machine outcomes.

**Fig. 4:**
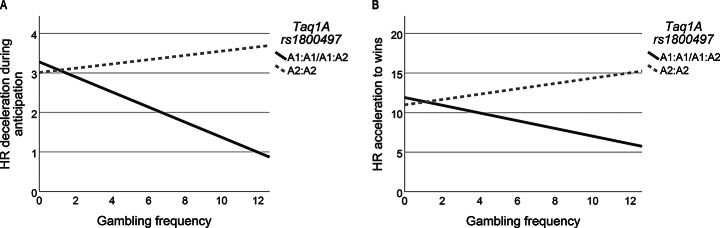
Illustration of GxE interactions on HR responses **A**) Interactions between Taq1A variants and previous gambling frequency on HR deceleration during anticipation. **B**) Interactions between Taq1A variants and previous gambling experience on HR acceleration to wins

Although there were no significant interactions between Taq1A variants and gambling frequency on anticipatory HR deceleration responses when analyzing homozygous and heterozygous alleles separately, an interaction was revealed when analyzing the effect of A1:A1/A1:A2 versus A2:A2 alleles. Specifically, A1:A1/A1:A2 carriers showed decreased anticipatory HR deceleration responses with increased level of gambling frequency (*B* = -0.245, *CI =* -0.475 - -0.015, *p* = 0.037) (Fig. 4A).

Further, analyses showed a significant interaction between Taq1A variants and gambling frequency on HR acceleration responses to wins (*p* = 0.036). Although, mean comparisons indicated the largest HR acceleration to wins with increased gambling frequency among A2:A2s, followed by A1:A2s, and A1:A1s. However, post hoc analysis only indicated significantly decreased HR acceleration to wins with increased level of gambling frequency in A1:A2 carriers compared to A2:A2s (*B* = -0.723, *CI* = -1.421– -0.026, *p* = 0.042). Interaction effects of A1:A1s failed to reach significance, potentially due to low levels of gambling frequency in this group. Analyzing the effects of A1:A1/A1:A2 versus A2:A2 carriers showed decreased HR acceleration to wins with increased level of gambling frequency among A1:A1/A1:A2 carriers (*B* = -0.825, *CI* = -1.516– -0.135, *p* = 0.019) (Fig. 4B). No further interactions were shown between Taq1A variants and HR responses to slot machine outcomes. Figure 4A and B illustrate HR responses in *dichotomized groups* of Taq1A alleles.

#### *DRD2*, C957T (rs6277)

There were no significant interactions between C957T (rs6277) variants and gambling frequency on SCRs, HR deceleration or acceleration responses during anticipation or to different slot machine outcomes.

## Discussion

This study investigated the associations between polymorphic variants regulating D2 dopamine receptor expression and autonomic responses during a slot machine gambling task, and whether these putative associations were modulated by sex and the degree of exposure to gambling, in a community sample of young adults. Results revealed differential relationships between Taq1A (rs1800497) and C957T (rs6277) allelic variants and ANS processing during both anticipation and outcome delivery depending on the type of psychophysiological measurement used to define autonomic responses. Overall, an increasing level of gambling frequency was associated with reduced reward responses, as indexed by SCRs. Furthermore, cG×E interactions were seen between the Taq1A genotype and gambling frequency on HR responses during anticipation and to wins. These results suggest that the investigated genotypes may be associated with differential ANS sensitivity to slot machine gambling cues and rewards.

### Associations Between Genotypes and ANS Responses

Variants of both Taq1A and C957T have shown to cause substantial changes in the neural regulation of D2 receptor density (Gluskin & Mickey, 2016; Hirvonen et al., 2004, 2005, 2009; Noble et al., 1991; Pohjalainen et al., 1998; Ritchie & Noble, 2003; Thompson et al., 1997). Based on our results, we suggest that these variants may also relate to differential ANS responses, thought to index emotional responsivity in gambling. The physiological measures used are known to index differential psychophysiological processes. Hence, the combined analysis of different ANS components (SCRs, HR deceleration and acceleration) enabled analyses on the dopaminergic influences on different aspects of affective processing. The HR deceleration response reflects an initial orienting response associated with perceptual processing that supports attentional processes. The subsequent HR acceleration response, as well as SCRs, are both primarily indicative of arousal and emotional reactivity (Bradley et al., 2001; Codispoti et al., 2001; Lang et al., 1993). Regarding the Taq1A polymorphism, previous research has proposed reduced reward sensitivity in carriers of the A1 variant, due to desensitization of the D2 receptors (Cohen et al., 2005). The direction of these effects on ANS measures reported in this study seems to contradict these reports. Results of the present study regarding the association between genotypes and SCR responses revealed that carriers of the Taq1A A1 allele showed increased emotional responsivity during anticipation (indicated by SCRs) than carriers of only the A2 allele. A1 carriers showed greater reward responsivity (indicated by SCRs) to win outcomes, compared with A2 homozygotes. Hence, the presence of the A1 allele, related to reduced receptor density, was associated with increased physiological reward and anticipatory responses compared with A2 homozygotes, in the overall sample.

Interpretations regarding the mechanism of C957T are less straightforward given that effects in this context appear driven by heterozygosity of this polymorphism. Male heterozygotes showed increased emotional responsivity during anticipation, as well as to full-misses (indicated by SCRs) compared to male C homozygotes. These relationships were not evident when comparing the presence of the T allele with homozygous C carriers, which may relate to differences in functionality of this genotype in different brain areas. The C allele has been associated with reduced D2 receptor density in the striatum (Hirvonen et al., 2004, 2005), but higher D2 receptor availability in the cortex and thalamus (Hirvonen et al., 2009). Further research is needed to disentangle the functional mechanism by which these changes may be manifested through ANS responses. The role of D2 receptors and the DRD2 gene have been implicated in addiction and reward related disorders, but the precise functional mechanisms of Taq1A and C957T allelic variants in emotional and cognitive regulation and their resulting behavioral phenotypes are currently undetermined. It is possible that effects of these variants may be more apparent in interactions with other genotypes, especially concerning the C957T polymorphism. Due to its proximity to the Taq1A locus, previous association studies have investigated haplotypes including both C957T and Taq1A as genetic basis for D2 receptor expression (Doehring et al., 2009; Hirvonen et al., 2009). One experimental study by Richter et al. (2017) found that both Taq1A A1 carriers and C957T C homozygotes showed better memory for rewarding stimuli during an incentive delay task followed by a delayed memory test.

However, a central aspect regarding the difficulty of reconciling the results with previous studies reporting effects of dopaminergic genes on reward mechanisms, is the nature of reward context especially within a gambling framework. The literature on the dopaminergic neural basis of reward-related and gambling behavior is characterized by heterogeneity of tasks, which often include a variety of cognitive elements of executive function, decision-making, learning, delay-discounting etc. A central aspect to reward processing in gambling is that more salient stimuli capture attention and more powerful expectancy effects and reward responses (Clark et al., 2019; Limbrick-Oldfield et al., 2017; Sescousse et al., 2013; van Holst et al., 2012). Slot machines are considered one of the most addictive forms of gambling (Binde et al., 2017; Markham et al., 2015), however, the cues and outcomes of slot machine gambling likely engage different cognitive and affective processes, than the tasks used in other studies. For example, due to the relatively repetitive nature of this form of gambling the anticipatory phase may not necessarily correspond to the ones seen in tasks requiring a higher level of skill or risk-taking. The anticipation and reward stimuli in slot machines are also accompanied by random contingencies of near-misses which are known to elicit large physiological responses, inflated reward expectancy and motivational effects (Billieux et al., 2012; Clark et al., 2009, 2012a; Cote et al., 2003; Dixon et al., 2011; Hultman et al., 2022; Kassinove & Schare, 2001; MacLin et al., 2007). Increased responses following near-misses have previously been reported in the current study sample, showing that ANS responses to near-misses were characterized by larger SCRs than full-misses and larger HR acceleration than both wins and full-misses (Hultman et al., 2022). However, the current study did not detect any differences in responses to near-misses across Taq1 or *C957T* genotypes.

As described, individual differences were evident in terms of sex. Generally, the effects of Taq1A/C957T variants were prominent among males. These were characterized by larger anticipatory responses in male Taq1A A1:A1/A1:A2s and C957T heterozygotes, larger responses to wins in male Taq1A A1 homozygotes, and larger responses to full-misses among male C957T heterozygotes. Some evidence reports sex differences in D2 receptors, suggesting lower D2 dopamine-binding potentials in striatal regions among females, and a role of estrogen in modulating dopamine release and dopamine D2 gene expression. However, sex differences in reward sensitivity are currently inconclusive and may be driven by a complex interplay between genetic, hormonal, environmental, developmental, and socialization factors [see Diekhof (2018) for a review].

It is possible that the differences observed in the current study are related to inherent sex differences in the expression of dopaminergic allelic variants. It may also result from sex differences in the level of gambling experience, due to the significantly higher level of gambling frequency among males in the current sample.

#### cG×E Effects of Genotypes and Gambling Exposure

The correlation between reduced ANS responsivity to rewards and gambling frequency, are in accordance with studies reporting blunted reward responses among frequent gamblers (Balodis et al., 2012; Choi et al., 2012; de Ruiter et al., 2009; Reuter et al., 2005), but similar responses during the anticipation phase were not observed. Due to the relatively low level of gambling frequency in the current sample, main overall effects of genotypes reflect the perceptual value of gambling stimuli among most participants with none, to moderate gambling frequency overall. Although main effects of genotypes were characterized by increased autonomic affective responses during anticipatory and outcome phases, cG×E effects were characterized by an inverse relationship for Taq1A x gambling frequency on HR responses during both anticipation and to winning outcomes. Individuals heterozygous or homozygous for the A1 allele generated decreased HR deceleration during anticipation and decreased HR acceleration to wins with higher level of gambling frequency, suggesting that higher gambling frequency among heterozygous or homozygous A1 carriers may lead to less attention orienting during anticipation and reduced reward responsivity in slot machine gambling.

Theories suggesting reward deficiency in gamblers posit that repeated exposure to the addictive reinforcer leads to *blunted pleasurable effects of rewards* over time, while theories on incentive-sensitization further suggest that repeated exposure leads to perceptual escalation of incentive salience of *appetitive cues associated with the addictive reinforcer*, promoting sensitization of the dopamine system (Berridge & Robinson, 2016; Robinson & Berridge, 1993, 2000, 2008). The result of this study suggests that these effects may be especially prominent in subgroups carrying dopaminergic genotypes resulting in reduced dopamine D2 receptor availability and thereby contributing to differential sensitivity to gambling cues in relation to previous exposure to gambling. These results add to previous findings indicating an inverse relationship between striatal recruitment and gambling related impairments (Balodis et al., 2012). The inverse relation between gambling experience and ANS responses during both anticipation and rewards in Taq1A A1 carriers may also reflect less incentive salience to simplified slot machine cues in this subgroup. However, such interpretations need to be substantiated by highly powered studies providing samples with a more even distribution of gamblers across allelic variants. Notably, the overall level of gambling frequency among the subgroup of gamblers was relatively low. Thus, it is possible that a stronger link between dopaminergic genotypes and higher gambling exposure on ANS responses would be apparent in a well-defined subgroup of frequent gamblers.

No cG×E effects were observed for ANS responses to near-misses. Studies on the dopaminergic activity associated with stimuli signaling the absence of reward and near-misses in gamblers, are currently lacking. In Sescousse et al. (2016) the amplified striatal activity to near-misses observed in gamblers was not significantly modulated by a dopamine D2 receptor antagonist. However, Worhunsky et al. (2014) reported blunted striatal activations to near-misses and loss outcomes in gamblers compared to non-gamblers, suggesting that repeated experiences of near-misses may decrease responses in dopamine-rich brain regions over time (Worhunsky et al., 2014). As previously stated, further studies on dopaminergic functions in relation to emotional responsivity to near-misses and losses in highly powered studies including well-defined groups of gamblers would provide well needed perspectives on the individual affective processing of structural characteristics known to promote continuous gambling.

### Strengths and Limitations

This study included a relatively large sample of young adults for this kind of experimental setting, with an evenly distributed subset of males and females, recruited from a community-based cohort. Complementary measures of SCRs and HR responses provide additional knowledge regarding the impact of individual differences in dopaminergic regulation on differential psychophysiological processes during slot machine gambling.

Still, the limitations of the study mainly concern power issues in the cG×E analysis, due to the small proportion of participants with any gambling exposure. The exclusion of participants due to technical failure in the EDA recording may have reduced statistical power and introduced bias, especially in the interaction models. Notably, cG×E effects were only observed in the HR response measures but not in terms of SCRs, suggesting insufficient power due to the exclusion of a large number of participants in the SCR analyses, including a number of participants with previous gambling experience. This likely introduced some bias in the cG×E models regarding the SCRs. Since this study included a community-based sample of young adults, the level of gambling among the subgroup of participants with gambling experience was also relatively low, which restricts the generalizability of the results to populations of more frequent or problem gamblers. Also, no definitive conclusions regarding sex differences can be made, due to the significantly higher level of previous gambling among males in the current sample.

This study conducted multiple testing given several outcome variables of interest. Still, crude p-values were applied due to the exploratory nature of this study. While corrections for multiple testing are critical in confirmatory studies to control the experiment-wise error rate and ensure valid conclusions, the resulting reduction of statistical power following such corrections would be counterproductive in an exploratory setting such as the current study (Bender & Lange, 2001). Considering this, associations between genotypes and ANS responses as well as sex and cG×E interactions was indeed detected. Thereby, this study provides an indication that dopaminergic genetic markers may have an impact on differential emotional ANS processing to slot machine stimuli depending on sex and previous gambling, but this need to be substantiated by highly powered studies.

A critical aspect is that the investigated loci are not the only ones affecting the dopaminergic system. A stronger association between genes related to dopaminergic neurotransmission and ANS responses during gambling may be even more apparent when testing interactions between several polymorphisms, rather than separate alleles. Polymorphisms in genes related to other monoaminergic functions have also been implicated in reward responses and gambling behavior (Courtiol et al., 2021; Leeman & Potenza, 2013), with research suggesting that neurotransmitters such as serotonin, glutamate and GABA influence reward- and loss-processing (Leeman & Potenza, 2012). The potential additive effects of multiple candidate genes involved in dopamine, serotonin, norepinephrine, and GABA functioning have also been suggested (Comings et al., 2001). Hence, a direction for future research would be to investigate interactions between several gene variants on ANS responses during gambling. Furthermore, dopaminergic signaling and ANS responses may be modulated by other environmental influences, psychiatric diagnoses, neuropsychiatric disabilities, hormonal levels, or other undetermined factors, which were not controlled for in this study.

This study used a slot machine task previously shown to reliably capture distinct phasic responses to different gambling stimuli (Clark et al., 2012a; Hultman et al., 2022; Sescousse et al., 2016), reinforcing the validity of a laboratory setting in detecting distinct emotional reactions to gambling-related stimuli. However, a computerized slot machine task in a laboratory setting is indeed simplified compared with real-life slot machines, which typically feature multiple reels and sequential symbol stops which may increase anticipation compared to two-reel tasks (Dixon et al., 2010; Palmer et al., 2024). Therefore, a simplified laboratory gambling task may not fully capture the emotional intensity of real-life gambling (Winstanley et al., 2016). It was also part of a larger experimental session in which the participants performed several other tasks. Hence, the potential effects of boredom and disinterest cannot be ruled out.

## Conclusion

To the best of our knowledge, this is the first study reporting a relationship between dopaminergic genotypes and ANS responses during anticipation and to outcomes in a slot machine task delivering unpredictable wins, near-misses and full-misses, while also considering previous gambling exposure and sex differences within a community sample of young adults. The results suggest that polymorphic variants related to dopaminergic neural regulation may confer differential ANS response sensitivity to gambling stimuli. Most prominently, genetic predispositions toward lower D2 receptor density in Taq1A A1 carriers were associated with increased physiological reward and anticipatory responses. Greater ANS responsivity during anticipation and delivery of wins and full-misses was also observed in heterozygote variants of the C957T polymorphism. Considering exposure to gambling there was an inverse relationship between A1 carriers and gambling frequency on ANS responses during both anticipation and to winning outcomes. Sex differences were also evident and expressed differently depending on the investigated genotype and outcome measure.

To conclude, this study provides preliminary indications that functional markers of the D2 dopamine receptor may contribute to differential ANS sensitivity to slot machine gambling cues and rewards. Due to heterogeneity of the literature on the dopaminergic neural basis of reward-related behavior no definitive conclusions can be drawn regarding the influence of these polymorphisms on emotional processing during chance-based gambling. Further research is needed to disentangle the functional mechanism by which these changes are manifested through ANS responses and how they may operate in relation to environmental factors to promote appetitive behavior in a gambling context.

Nevertheless, results add to previous research on the dopaminergic neural basis of emotional processing of outcomes within the specific reward context of slot machine gambling, characterized by intermittent random reward delivery and near-misses, producing maximum outcome uncertainty. This may also be modulated by sex and exposure to gambling activities to some extent, but this conclusion requires further study in well-defined samples of gamblers and control subjects. Future studies should also aim to clarify the potential relationship between differential sensitivity during gambling and the risk of developing problematic gambling behavior.

## Data Availability

The data used in the research cannot be publicly shared due to ethical or legal restrictions but are available upon request.
